# Increased frontal brain activation during walking while dual tasking: an fNIRS study in healthy young adults

**DOI:** 10.1186/1743-0003-11-85

**Published:** 2014-05-12

**Authors:** Anat Mirelman, Inbal Maidan, Hagar Bernad-Elazari, Freek Nieuwhof, Miriam Reelick, Nir Giladi, Jeffrey M Hausdorff

**Affiliations:** 1 Movement Disorders Unit, Department of Neurology, Tel-Aviv Sourasky Medical Center, 6 Weizmann Street, Tel Aviv 64239, Israel; 2 RiVERS Lab, Department of Rehabilitation and Movement Science, University of Medicine and Dentistry of New Jersey, Newark, USA; 3 Sagol School of Neuroscience, Tel Aviv University, Tel Aviv, Israel; 4 Department of Neurology, Sackler Faculty of Medicine, Tel-Aviv University, Tel-Aviv, Israel; 5 Department of Medicine, Harvard Medical School, Boston, MA, USA; 6 Department of Physical Therapy, Sackler Faculty of Medicine, Tel-Aviv University, Tel-Aviv, Israel; 7 Departments of Geriatric Medicine and Neurology, Radboud University Nijmegen Medical Center, Nijmegen, The Netherlands; 8 Radboud University Nijmegen Medical Centre, Donders Institute for Brain, Cognition and Behavior, Nijmegen, The Netherlands

**Keywords:** Gait, Dual task, Frontal lobe, Imaging, fNIRS

## Abstract

**Background:**

Accumulating evidence suggests that gait is influenced by higher order cognitive and cortical control mechanisms. Recently, several studies used functional near infrared spectroscopy (fNIRS) to examine brain activity during walking, demonstrating increased oxygenated hemoglobin (HbO_2_) levels in the frontal cortex during walking while subjects completed a verbal cognitive task. It is, however, still unclear whether this increase in activation was related to verbalization, if the response was specific to gait, or if it would also be observed during standing, a different motor control task. The aim of this study was to investigate whether an increase in frontal activation is specific to dual tasking during walking.

**Methods:**

Twenty-three healthy young adults (mean 30.9 ± 3.7 yrs, 13 females) were assessed using an electronic walkway. Frontal brain activation was assessed using an fNIRS system consisting of two probes placed on the forehead of the subjects. Assessments included: walking in a self-selected speed; walking while counting forward; walking while serially subtracting 7s (Walking+S7); and standing while serially subtracting 7s (Standing+S7). Data was collected from 5 walks of 30 meters in each condition. Twenty seconds of quiet standing before each walk served as baseline frontal lobe activity. Repeated Measures Analysis of Variance (RM ANOVA) tested for differences between the conditions.

**Results:**

Significant differences were observed in HbO_2_ levels between all conditions (p = 0.007). HbO_2_ levels appeared to be graded; walking alone demonstrated the lowest levels of HbO2 followed by walking+counting condition (p = 0.03) followed by Walking+S7 condition significantly increased compared to the two other walking conditions (p < 0.01). No significant differences in HbO_2_ levels were observed between usual walking and the standing condition (p = 0.38) or between standing with or without serial subtraction (p = 0.76).

**Conclusions:**

This study provides direct evidence that dual tasking during walking is associated with frontal brain activation in healthy young adults. The observed changes are apparently not a response to the verbalization of words and are related to the cognitive load during gait.

## Background

The relationship between gait and executive function has been explored using behavioral testing, neuroimaging, and other indirect methods [1-3]. Executive function and attention, cognitive domains that reflect frontal lobe function, are associated with gait performance [4-7]. Slower gait was related to smaller prefrontal volumes in older adults [8,9], and gray matter abnormalities in cognitive regions were associated with gait alterations [10]. Moreover, behavioral studies demonstrate that walking while carrying out a dual task (DT) decreases gait speed and increases dysrythmicity [2,11]. Interestingly, even healthy young adults may slow down when they are asked to walk and perform a relatively challenging secondary DT simultaneously [11]. Findings from these three lines of research all suggest that gait relies on cognitive resources, especially those associated with frontal and pre-frontal lobe activation.

Recently several studies used fNIRS to examine brain activity during walking [12,13]. NIRS measures use optical absorption to detect hemodynamic changes in the prefrontal cortex [14], similar to the way that activation is assessed using magnetic resonance imaging. Optodes placed on the surface of the forehead send and receive light (wavelengths 750 to 1000 nm), recording the changes in returning light that has traversed through the skull. The rationale behind fNIRS is that relevant stimuli produces an increase in regional cerebral blood flow due to higher energy demands in “activated” areas, resulting in higher blood oxygenation. Using fNIRS, an increase in oxygenated hemoglobin (HbO_2_) levels in the frontal cortex was reported during walking while talking in both healthy young and older adults [13]. A similar, but smaller effect was seen in older adults with mild cognitive impairment [12,13]. These changes in HbO_2_ provide evidence for cortical involvement during dual task gait even in healthy young adults. It is, however, not clear if these HbO_2_ changes during walking while talking are due to the effect of verbalization, the dual task effect, the demands of gait, or if it simply reflects a process that would also be observed during other motor tasks like standing.

The present study addressed these issues to better understand the dependence of gait on frontal lobe function and the effects of a simple and a more cognitively demanding DT on gait. We hypothesized that frontal lobe activation would be higher during walking with a complex DT, as compared to walking with a simple DT resulting in higher levels of HbO_2_.In addition we further hypothesized that walking would result in higher HbO_2_ levels than standing in both simple and complex conditions.

## Methods

### Participants

Twenty-three healthy young adults (mean 30.9 ± 3.7 yrs; range: 24-38 yrs, 13 females; years of education: 17.7 ± 2.4) were studied. Subjects were included if they were healthy, had no underlying orthopedic or neurological disorders, and were cognitively intact based on the Montreal Cognitive Assessment score (>26) [15]. All participants provided informed written consent as approved by the local ethics committee, the Helsinki committee at Tel Aviv Medical Center.

### Procedures

Subjects performed four tasks while instrumented with two fNIRS probes in the following fixed order: 1) walking in a self-selected comfortable speed (usual-walking), 2) walking while counting forward (to control for verbalization and task complexity: walking+counting), 3) walking while serially subtracting 7 s from a pre-defined 3-digit number (a complex DT: walking+S7), and 4) standing in place while serially subtracting (standing+S7). Each walking task consisted of five walks of 30 meters in a stimuli free hallway. The standing task included five S7 intervals of 30 seconds each. There was 20 seconds of quiet standing before and after each of the walking and standing+S7 tasks.

### Assessment of frontal lobe function

Frontal brain activation was assessed using an fNIRS system (Artinis, The Netherlands). Subjects were instrumented with two separate pairs of NIRS probes each containing 3 transmitters for a total of 6 channels. The NIRS transmitter and receiver pairs were placed over the left (Fp1) and right (Fp2) frontal cortex regions of the forehead, according to the modified international EEG 10-20 system at a height of 15 % of the distance from nasion to inition and at 7 % of head circumference from left and right. Micromolar changes in HbO_2_ and deoxyhaemoglobin (HHb) were determined using received optical densities from two continuous wavelengths of NIR light (760 and 850 nm). Task related changes in HbO_2_ were evaluated relative to the quiet standing HbO_2_ level before each task to control for transient effects of the signal [12,13]. For the walking and standing+S7 conditions, the average level of HbO_2_ during the immediately preceding 20 seconds of quiet standing was subtracted from the levels of HbO_2_ observed during the task; the average across the 5 repetitions of each task summarized the value in each condition. fNIRS data was recorded continuously at 10 Hz. The NIRS depth signal was determined as half of the distance from transmitter to detector [16]. This depth corresponds to the superficial Brodmann 10 area as determined by a previous MRI (TI sequence) study in healthy adults in which the location of the fNIRS probes were marked.

### Assessment of gait

A sensorized 7 meter carpet (PKMAS) captured individual footfall data (at 120 Hz) to determine spatiotemporal gait characteristics (e.g., gait speed) and stride-to-stride variability [17,18], a measure of gait inconsistency associated with executive function. Stride time variability was determined using the coefficient of variation (CV).

### Data analysis

To eliminate physiologically irrelevant effects, a low-pass filter was applied with a finite impulse response filter, with a cutoff frequency at 0.14 Hz before processing the signals. HbO_2_ concentration was calculated as the average of the combined 6 channels from both sides of the forehead as no differences were observed between the hemispheres. HbO_2_ values were chosen to characterize the tasks as they are more reliable and sensitive to locomotion-related cerebral blood flow than HHb values [19]. Continuous Wavelet transform (CWT) is a method of measuring the cross-correlation between 2 time series as a function of frequency, intensity and time of change in the wavelet [20,21]. CWT compared the fNIRS signals during usual-walking and walking+S7 using the wavelet coherence Matlab package [20]. The wavelet coherence was conducted within each condition and then a comparison of wavelet coherence analyses was made between conditions.

HbO_2_ and the gait data were examined for homogeneity using box and scatter plots; means and standard deviations are presented. Repeated Measures ANOVA was used to assess differences between conditions in HbO2 levels and gait measures. Post-hoc assessment was tested for differences between the tasks in both gait and HbO_2_. Significance levels were set at 0.05.

## Results

As expected, gait speed, stride length and stride time differed between the different walking conditions (p < 0.0001). Gait speed during walking+S7 was slower and gait variability tended to be higher (less consistent) than during usual-walking (Table 1). The number of subtractions completed was similar during walking+S7 and standing+S7 (10.5 ± 0.5 vs.12.0 ± 0.6 respectively; p = 0.91) with a similar number of mistakes (p = 0.89).

**Table 1:** Gait in the different conditions

	**Usual walking**	**Walking+counting**	**Walking+S7**	**P-value**
Gait speed (m/s)	1.35 ± 0.1	1.29 ± 0.15	1.23 ± 0.14*	<0.0001
Stride length (m)	1.45 ± 0.12	1.45 ± 0.11	1.39 ± 0.11*	<0.0001
Stride time (s)	1.09 ± 0.08	1.13 ± 0.08	1.14 ± 0.07*	<0.0001
Stride time variability (%)	2.35 ± 0.50	2.26 ± 1.28	2.47 ± 1.04	0.777

*significant difference between usual-walking and walking + S7 in post-hoc analysis.

**Figure 1:**
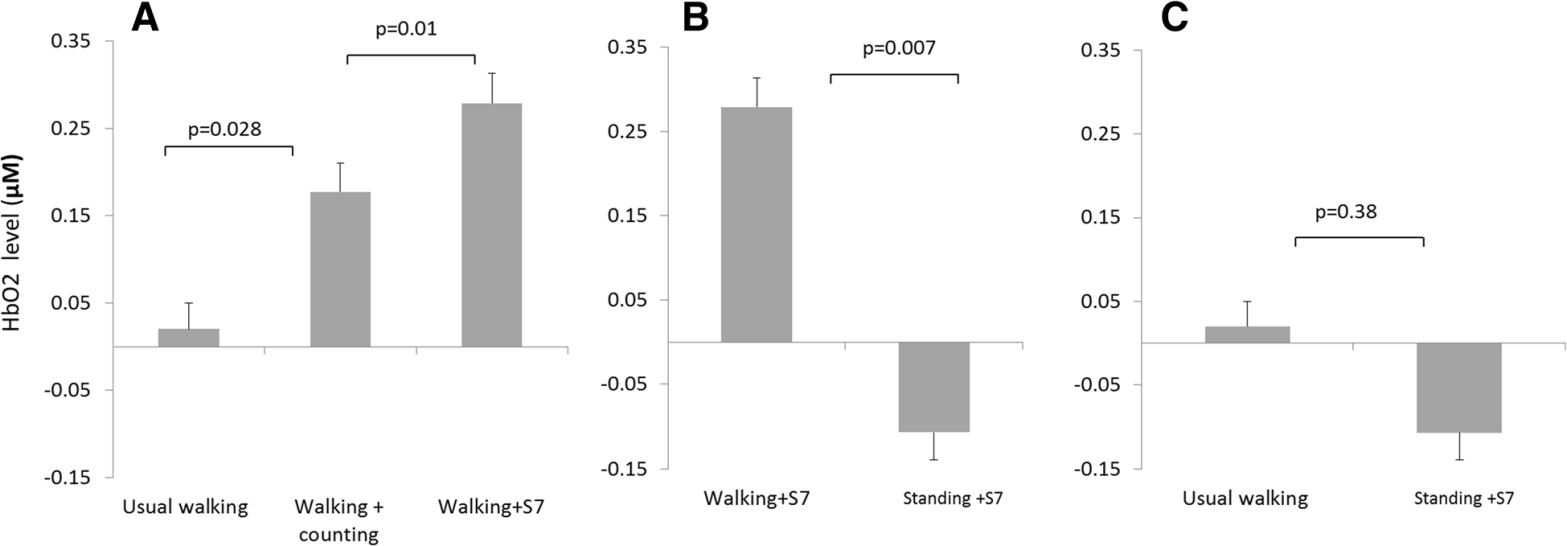
**HbO**_**2 **_**levels (mean±SE are shown) responded with a graded response to task complexity.** Compared to quiet standing, the highest increase was observed in the walking+S7 condition, a moderate increase in the walking and counting condition, and the lowest increase in the usual-walking **(A)**. Differences were observed in HbO_2_ response between DT conditions in standing and walking **(B)** and between simple counting task in walking and standing **(C)**. P values represent the post-hoc analysis between conditions.

**Figure 2:**
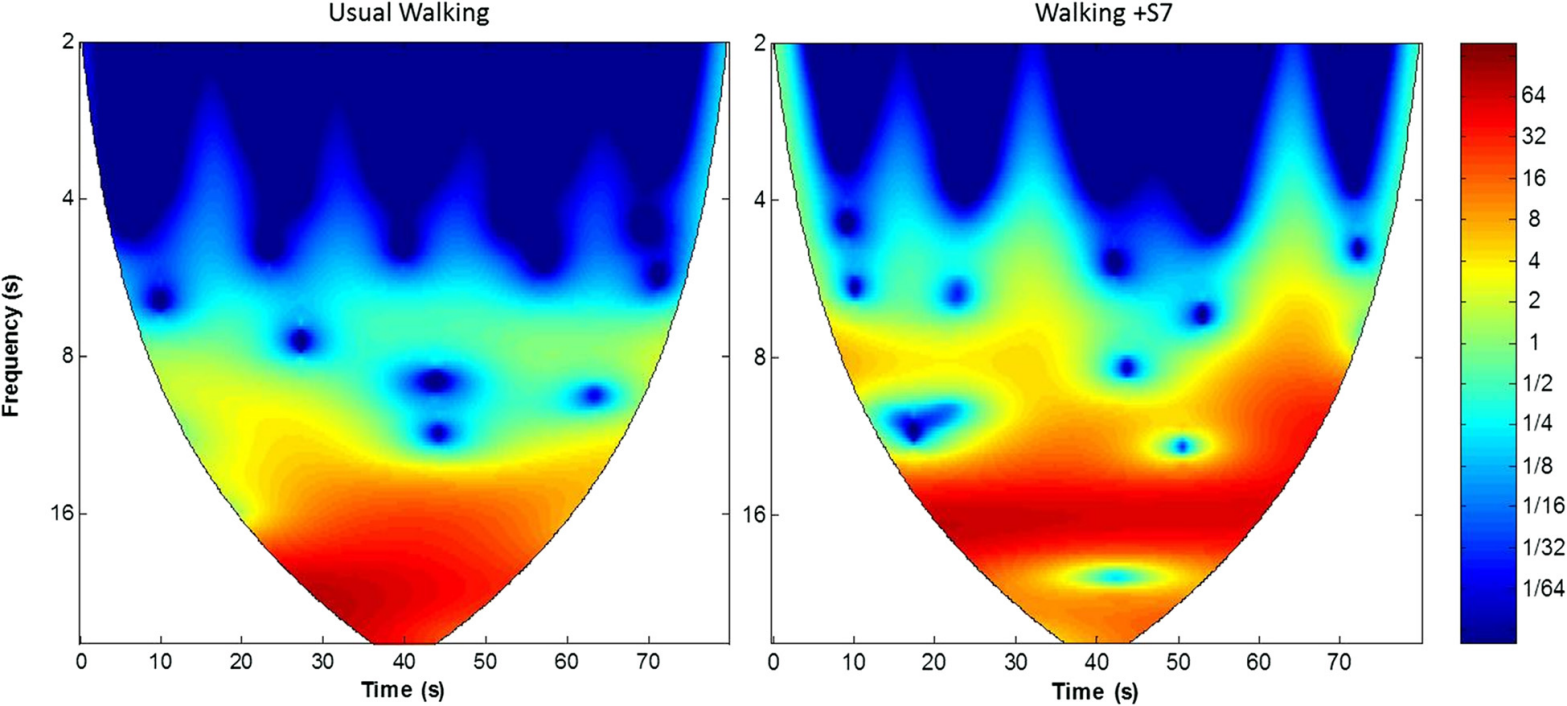
**Continuous wavelet transform of time series in different tasks: (Left) usual-walking, (Right) walking+S7.** The raw time courses of the task were analyzed in the frequency domain to express the oxygenation response in terms of intensity (concentration of HbO_2_ levels) and timing (slow or rapid response in HbO_2_ levels). An inclination towards more oxygenation (represented by warmer colors) was observed around 20-30 frequency seconds in usual-walking, suggesting low frequencies (relatively slow change) in HbO_2_ signal as compared to quiet standing. In contrast, during walking+S7, a stronger and more rapid oxygenation pattern (represented by warm colors) appears earlier (12-20 frequency seconds), reflecting a quick and intense increase in oxygenation.

HbO_2_ levels did not differ (p > 0.169) across the repeated tests of quiet standing. Significant differences were observed in HbO_2_ levels between all task conditions, responding to the complexity of the DT with a graded increase (p = 0.007). Compared to quiet standing, the highest increase was observed during walking+S7, a moderate increase was seen during walking + counting, and the lowest, non-significant increase was observed during usual-walking (see Figure 1A). Compared to the usual-walking task, HbO_2_ levels increased by 0.15 μM (p = 0.03) during walking+counting and by 0.26 μM in the walking+S7 condition (p = 0.009) (Figure 1A). HbO_2_ levels also differed between walking+counting and walking+S7 (p = 0.01) and walking+S7 and standing+S7 (p = 0.007) (Figure 1B). No differences were observed in HbO_2_ levels between the usual-walking condition and standing+S7 (p = 0.38) (Figure 1C) or between standing+S7 and quiet standing (p = 0.76). The spectrogram analysis of the CWT revealed that the frequency and intensity of oxygenation during the walking+S7 task differed from that observed during usual-walking (Figure 2).

**Figure 3:**
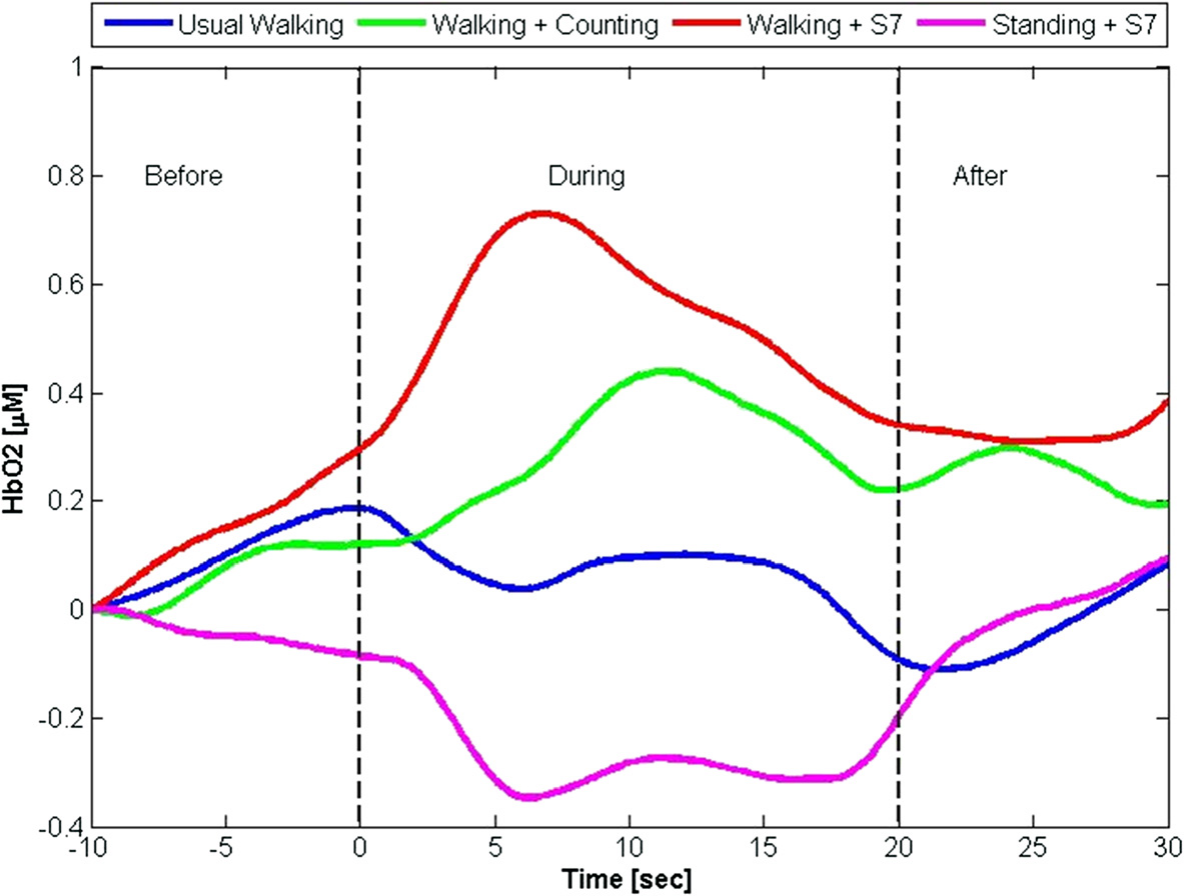
**The time line represents the change in HbO**_**2 **_**from quiet standing (before), during the task, and the return to a steady resting hemodynamic state after the completion of the task (after).** HbO_2_ level increases dramatically as a result of the challenging cognitive task during walking (red line). During usual-walking (blue line), levels of HbO_2_ remained close to quiet standing levels. HbO_2_ increased in the walking and counting condition (green line), however, there was a delayed response, suggesting that the task did not affect activation of the PFC. Standing+S7 (pink line) demonstrated a delayed HbO_2_ decrease during the task. All measures returned towards pre-task values consistent with a hemodynamic response.

Gait speed, stride length and stride time were not correlated with HbO_2_ levels in any of the walking conditions. During walking+S7, gait variability was inversely associated with HbO_2_ levels (r = -0.47, p = 0.04). HbO_2_ levels during this task were also inversely correlated to the number of subtractions that were completed during this walk (r = -0.71, p = 0.011). This association was not observed in the standing+S7 condition (p = 0.53) or the walking+counting condition (p = 0.35). HbO_2_ levels changed during the tasks and returned to quiet standing levels during the resting period after the tasks (Figure 3), consistent with a physiologic hemodynamic response to a functional task.

## Discussion

The current results showed that dual tasking increased brain activation in prefrontal areas during walking in young adults. We observed a graded response as a function of the cognitive demand during walking. Oxygenation levels were higher during walking+counting, compared to usual-walking, and were even higher during walking+S7, a more cognitively demanding, complex DT. In contrast, standing+S7 lead to a decrease in blood oxygenation levels. Taken together, these findings suggest that frontal activation during walking while carrying out a DT is not solely a reflection of verbalization and is very different from the response observed during quiet standing or during standing while DT.

The present findings build on several earlier reports that explored the use of fNIRS to assess DT walking [12,13]. The current results extend the previous findings by demonstrating the impact of the complexity of the DT and, as discussed above, by the increase in blood oxygenation levels in the PFC seen during walking but not standing. The direction of increase of HbO_2_ between single and DT walking was similar in our study to that observed by Doi et al [12], although the younger participants showed slightly higher baseline levels of activation than the patients with mild cognitive impairment. Thus, it is likely that oxygenation levels in response to a DT during walking may be affected by age and disease, decreasing the initial activation level and limiting the hemodynamic response and the ability of the brain to adapt to complex situations. This is consistent with the hypotheses explaining possible mechanisms of diminished DT performance in older adults [22,23]; future studies should further evaluate these issues.

One of the novelties of the present findings is that it demonstrates the DT effect is specific to gait, at least as compared to standing. The hemodynamic activation during a relatively complex motor-cognitive task is linked with neurovascular coupling and increased oxyHemoglobin [13,24]. Based on an earlier evaluation of the fNIRS placement using fMRI, the measures of HbO_2_ in our study are presumed to be generated by blood vessels in the rostral prefrontal cortex (PFC), i.e., Brodmann 10 (BA10). Lesions in the rostral PFC lead to deficits when patients need to coordinate the performance of a number of tasks (multi-tasking), or in ill-structured situations. Multitasking typically involves maintaining super-or sub-ordinate goals while performing another task [25-27]. Accordingly, lesions in the rostral PFC manifest as tardiness and disorganization, regardless of intellect and social skills [26-28]. Imaging studies of BA10 observed increased recruitment of the rostral parts of BA10 in multi-tasking conditions compared to a control task [27,29], consistent with the present and previous research in young adults [13,14]. Thus, the current finding of PFC activation during multitasking confirms the relationship between dual tasking and prefrontal lobe function, while also extending it, as detailed further below.

Cognitive distractions interfere with the control of gait and standing posture, perhaps by competing for the same pool of neural resources [4,30,31]. Previous reports on changes in postural control during standing with distractions suggest an impact of the cognitive load on the regulation of postural musculature [32,33].

Interestingly, our findings show that HbO_2_ levels were not different during quiet standing and usual-walking. Usual-walking apparently does not elicit a large increase in blood oxygenation in the frontal area as this could be considered a relatively simple motor task, as least in healthy young adults. Conversely, HbO_2_ levels were much higher when the walking task was paired with either simple counting or a more complex DT (S7). Based on these findings, one might conclude that walking itself does not impact Hb0_2_ levels, and the increases during counting and serial subtractions simply reflect the cognitive demands of these two tasks. However, since we observed a decrease in Hb0_2_ during standing+S7, this explanation is most likely incorrect. Rather, it appears that the motor-cognitive demands seen during walking carrying out another task elicit a specific response to DT, while quiet standing does not, even in healthy young adults.

We speculate that the somewhat surprising difference in the frontal activation patterns during walking+S7 and standing+S7 reflects disparities between the control mechanisms of standing balance and walking. Balance control is associated with attention, especially during a DT [34]. Attentional tasks are often associated with the DLPFC and specifically, Brodmann area 9 [35]. On the other hand, walking while dual tasking resulted in higher blood flow and oxygenation in the rostral frontal cortex (BA 10), an area which relates more to simultaneous processing of both motor and cognitive functions. Thus, the cognitive components of standing and walking, as reflected in Hb0_2_, are apparently task specific, consistent with a recent behavioral study [32]. Based on this interpretation, one can suggest that oxygenated blood was directed to task areas that were involved in balance during the cognitive task and due to the anatomical location of the DLPFC and the limitation of our measurement tool (assessing only the rostral frontal lobe), this activation was not observed. Another possible explanation could be attributed to the complexity of the combined tasks. Walking requires dynamic stability and control of the center of mass over a narrow base of support whereas standing requires more static balance control. The addition of the same cognitive task to these two motor tasks generated very different responses that may be related to task complexity and specific aspects of motor control. This disparity should be further explored in future studies.

Condition effects were observed in gait speed, stride length and stride time but not in gait variability. This finding coincides with previous reports [3,7,18]. Here we show, for the first time, that gait variability during DT was inversely correlated with HbO_2_ levels in the walking+S7 condition, indicating higher gait variability with lower HbO_2_ levels during DT. Executive function has been previously shown to be correlated with gait performance during DT [4]. This association is thought to reflect the complexity of information processing that may be explained by the bottleneck or capacity sharing theories [2,22,23], where the involvement of the two tasks creates a competition for overlapping resources even if performance remains intact and is presumably normal. Subjects in this study performed the cognitive task similarly during standing+S7 and walking+S7 yet the number of subtractions during walking+S7 was highly correlated to HbO_2_ levels. This inverse correlation indicated that individuals who performed better on the subtraction task or in essence found this task less difficult, had lower HbO_2_ levels than those who performed less well, who may need to recruit more cognitive resources to perform the task. In contrast, the cognitive task of subtraction did not correlate to oxygenation levels during the standing condition, perhaps because standing is a simpler, less complex task than walking and possibly the control of balance is related to other neural areas thus competition for the same resources might be smaller. This finding highlights the specific and unique relationship between gait and executive function and the increased involvement of overlapping cognitive resources during gait that do not appear in a simpler motor task.

This study has several limitations including the lack of an additional measurement in sitting to further explore the role of balance control and the limitation of our measurement tool. In addition, the order of the tasks was not randomized or counter-balanced which may serve as a limitation in other populations, however, in this young cohort we did not observe fatigue effects and HbO_2_ levels returned to baseline rapidly which we believe did not interfere with the measurements. The present findings strengthen the evidence from neuropsychological investigations on the connection between motor and cognitive function during walking in complex situations, while confirming and extending fNIRS studies of walking. The results of the present study could lead to the development of new strategies for training complex situations which are common in everyday life to improve dual tasking abilities [36]. Future studies should use a similar experimental design to assess healthy older adults and individuals with neurodegenerative disorders to assess differences in activation related to aging and disease.

## Abbreviations

fNIRS: functional near infrared spectroscopy; HbO_2_: Oxygenated hemoglobin; Walking+S7: Walking while serially subtracting 7 s from a 3 digit pre-defined number; Standing+S7: Standing in place while serially subtracting 7 s; CV: Coefficient of variation in gait (CV = standard deviation/mean x100).; CWT: Continuous wavelet transform; PFC: Pre frontal cortex; DLPFC: Dorso-lateral pre frontal cortex; DT: Dual task.

## Competing interests

The authors report no competing interests.

## Authors’ contributions

AM, JMH and IM designed the study. AM, IM, MR, FN designed the assessment protocol. IM and HBE collected the data and were involved in data processing and data analysis. Statistical analysis was performed by AM and JMH. The draft of the manuscript was prepared by AM and was critically reviewed by JMH, NG, IM, MR and FN. All authors confirmed and approved the final version of the manuscript.

## References

[B1] Amboni M Barone P Hausdorff JM Cognitive contributions to gait and falls: evidence and implications Mov Disord 2013 28 11 1520 1533 10.1002/mds.25674 24132840 PMC4119872

[B2] Yogev-Seligmann G Hausdorff JM Giladi N The role of executive function and attention in gait Mov Disord 2008 23 329 342 10.1002/mds.21720 18058946 PMC2535903

[B3] Hausdorff JM Yogev G Springer S Simon ES Giladi N Walking is more like catching than tapping: gait in the elderly as a complex cognitive task Exp Brain Res 2005 164 541 548 10.1007/s00221-005-2280-3 15864565

[B4] Hausdorff JM Schweiger A Herman T Yogev-Seligmann G Giladi N Dual-task decrements in gait: contributing factors among healthy older adults J Gerontol A Biol Sci Med Sci 2008 63 1335 1343 10.1093/gerona/63.12.1335 19126846 PMC3181497

[B5] Hausdorff JM Buchman AS What links gait speed and MCI with dementia? A fresh look at the association between motor and cognitive function J Gerontol A Biol Sci Med Sci 2013 68 409 411 10.1093/gerona/glt002 23401565 PMC3593618

[B6] Holtzer R Burright RG Donovick PJ The sensitivity of dual-task performance to cognitive status in aging J Int Neuropsychol Soc 2004 10 230 238 15012843 10.1017/S1355617704102099

[B7] Verlinden VJ van der Geest JN Hofman A Ikram MA Cognition and gait show a distinct pattern of association in the general population Alzheimers Dement 2013 doi: 10.1016/j.jalz.2013.03.009 10.1016/j.jalz.2013.03.009 23849591

[B8] Guo X Skoog I Matousek M Larsson L Palsson S Sundh V Steen B A population-based study on motor performance and white matter lesions in older women J Am Geriatr Soc 2000 48 967 970 10968303 10.1111/j.1532-5415.2000.tb06896.x

[B9] Rosano C Studenski SA Aizenstein HJ Boudreau RM Longstreth WT Jr Newman AB Slower gait, slower information processing and smaller prefrontal area in older adults Age Ageing 2012 41 58 64 10.1093/ageing/afr113 21965414 PMC3234076

[B10] Rosenberg-Katz K Herman T Jacob Y Giladi N Hendler T Hausdorff JM Gray matter atrophy distinguishes between Parkinson disease motor subtypes Neurology 2013 80 1476 1484 10.1212/WNL.0b013e31828cfaa4 23516323 PMC3662357

[B11] Yogev-Seligmann G Rotem-Galili Y Mirelman A Dickstein R Giladi N Hausdorff JM How does explicit prioritization alter walking during dual-task performance? Effects of age and sex on gait speed and variability Phys Ther 2010 90 177 186 10.2522/ptj.20090043 20023000 PMC2816029

[B12] Doi T Makizako H Shimada H Park H Tsutsumimoto K Uemura K Suzuki T Brain activation during dual-task walking and executive function among older adults with mild cognitive impairment: a fNIRS study Aging Clin Exp Res 2013 25 5 539 44 10.1007/s40520-013-0119-5 23949972

[B13] Holtzer R Mahoney JR Izzetoglu M Izzetoglu K Onaral B Verghese J fNIRS study of walking and walking while talking in young and old individuals J Gerontol A Biol Sci Med Sci 2011 66 879 887 21593013 10.1093/gerona/glr068 PMC3148759

[B14] Irani F Platek SM Bunce S Ruocco AC Chute D Functional near infrared spectroscopy (fNIRS): an emerging neuroimaging technology with important applications for the study of brain disorders Clin Neuropsychol 2007 21 9 37 10.1080/13854040600910018 17366276

[B15] Nasreddine ZS Phillips NA Bedirian V Charbonneau S Whitehead V Collin I Cummings JL Chertkow H The Montreal Cognitive Assessment, MoCA: a brief screening tool for mild cognitive impairment J Am Geriatr Soc 2005 53 695 699 10.1111/j.1532-5415.2005.53221.x 15817019

[B16] Muthalib M Anwar AR Perrey S Dat M Galka A Wolff S Heute U Deuschl G Raethjen J Muthuraman M Multimodal integration of fNIRS, fMRI and EEG neuroimaging Clin Neurophysiol 2013 124 2060 2062 10.1016/j.clinph.2013.03.018 23648071

[B17] Hausdorff JM Stride variability: beyond length and frequency Gait Posture 2004 20 304 10.1016/j.gaitpost.2003.08.002 15531178

[B18] Hausdorff JM Gait variability: methods, modeling and meaning J NeuroEng Rehabil 2005 doi:10.1186/1743-0003-2-19 10.1186/1743-0003-2-19 PMC1185560 16033650

[B19] Miyai I Tanabe HC Sase I Eda H Oda I Konishi I Tsunazawa Y Suzuki T Yanagida T Kubota K Cortical mapping of gait in humans: a near-infrared spectroscopic topography study Neuroimage 2001 14 1186 1192 10.1006/nimg.2001.0905 11697950

[B20] Grinsted A Moore JC Jevreheva S Application of the cross wavelet transform and wavelt coherence to geophysical time series Nonlinear Processes Geophys 2004 11 561 566 10.5194/npg-11-561-2004

[B21] Torrence C Compo GP A practical guide to wavelet analysis Bull Am Meterol Soc 1998 79 61 78 10.1175/1520-0477(1998)079<0061:APGTWA>2.0.CO;2

[B22] Ruthruff E Pashler HE Klaassen A Processing bottlenecks in dual-task performance: structural limitation or strategic postponement? Psychon Bull Rev 2001 8 73 80 10.3758/BF03196141 11340869

[B23] Tombu M Jolicoeur P A central capacity sharing model of dual-task performance J Exp Psychol Hum Percept Perform 2003 29 3 18 12669744 10.1037//0096-1523.29.1.3

[B24] Yamamoto T Kato T Paradoxical correlation between signal in functional magnetic resonance imaging and deoxygenated haemoglobin content in capillaries: a new theoretical explanation Phys Med Biol 2002 47 1121 1141 10.1088/0031-9155/47/7/309 11996059

[B25] Burgess PW Veitch E de Lacy CA Shallice T The cognitive and neuroanatomical correlates of multitasking Neuropsychologia 2000 38 848 863 10.1016/S0028-3932(99)00134-7 10689059

[B26] Burgess PW Strategy application disorder: the role of the frontal lobes in human multitasking Psychol Res 2000 63 279 288 10.1007/s004269900006 11004881

[B27] Okuda J Fujii T Ohtake H Tsukiura T Yamadori A Frith CD Burgess PW Differential involvement of regions of rostral prefrontal cortex (Brodmann area 10) in time- and event-based prospective memory Int J Psychophysiol 2007 64 3 233 46 10.1016/j.ijpsycho.2006.09.009 17126435

[B28] Shallice T Burgess PW Deficits in strategy application following frontal lobe damage in man Brain 1991 114 Pt 2 727 741 2043945 10.1093/brain/114.2.727

[B29] Dumontheil I Gilbert SK Frith CD Burgess PW Recruitment of lateral rostral prefrontal contex in spontaneous and task-related thoughts Q J Exp Psychol (Hove) 2010 63 1740 1756 10.1080/17470210903538114 20221947

[B30] Al-Yahya E Dawes H Smith L Dennis A Howells K Cockburn J Cognitive motor interference while walking: a systematic review and meta-analysis Neurosci Biobehav Rev 2011 35 715 728 10.1016/j.neubiorev.2010.08.008 20833198

[B31] Yogev-Seligmann G Giladi N Gruendlinger L Hausdorff JM The contribution of postural control and bilateral coordination to the impact of dual tasking on gait Exp Brain Res 2013 226 81 93 10.1007/s00221-013-3412-9 23371748

[B32] Dault MC Yardley L Frank JS Does articulation contribute to modifications of postural control during dual-task paradigms? Brain Res Cogn Brain Res 2003 16 434 440 10.1016/S0926-6410(03)00058-2 12706223

[B33] Kang HG Lipsitz LA Stiffness control of balance during quiet standing and dual task in older adults: the MOBILIZE Boston Study J Neurophysiol 2010 104 3510 3517 10.1152/jn.00820.2009 20844110 PMC3007648

[B34] Maki BE McIlroy WE Cognitive demands and cortical control of human balance-recovery reactions J Neural Transm 2007 114 1279 1296 10.1007/s00702-007-0764-y 17557125

[B35] Daffner KR Mesulam MM Scinto LF Acar D Calvo V Faust R Chabrerie A Kennedy B Holcomb P The central role of the prefrontal cortex in directing attention to novel events Brain 2000 123 Pt 5 927 939 10775538 10.1093/brain/123.5.927

[B36] Anguera JA Boccanfuso J Rintoul JL Al-Hashimi O Faraji F Janowich J Kong E Larraburo Y Rolle C Johnston E Gazzaley A Video game training enhances cognitive control in older adults Nature 2013 501 97 101 10.1038/nature12486 24005416 PMC3983066

